# Supplementary material for: Polarized microtubule remodeling transforms the morphology of reactive microglia and drives cytokine release
Source: Nat Commun. 2023 Oct 9;14:6322. doi: 10.1038/s41467-023-41891-6 (PMC10562429; doi:10.1038/s41467-023-41891-6)
Supplement: Supplementary file 1 — Supplementary Information [file 41467_2023_41891_MOESM1_ESM.pdf]

Polarized microtubule remodeling transforms the morphology of reactive microglia and drives cytokine release

Supplementary Information

Max Adrian[1,2], Martin Weber[1], Ming-Chi Tsai[1], Caspar Glock[3], Olga I. Kahn[1], Lilian Phu[4], Tommy K. Cheung[4], William J. Meilandt[1], Christopher M. Rose[4], Casper C. Hoogenraad[1, 5, *]

[1] Department of Neuroscience, Genentech, Inc., South San Francisco, CA 94080, USA
[2] Department of Pathology, Genentech, Inc., South San Francisco, CA 94080, USA
[3] Department of OMNI Bioinformatics, Genentech Inc., South San Francisco, CA 94080, USA
[4] Department of Microchemistry, Proteomics and Lipidomics, South San Francisco, CA 94080, USA
[5] Lead contact
* Correspondence to:
Casper C. Hoogenraad, Department of Neuroscience, Genentech, Inc., 1 DNA Way, South San Francisco, CA 94080, hoogenraad.casper@gene.com

Supplementary Figures 1-5

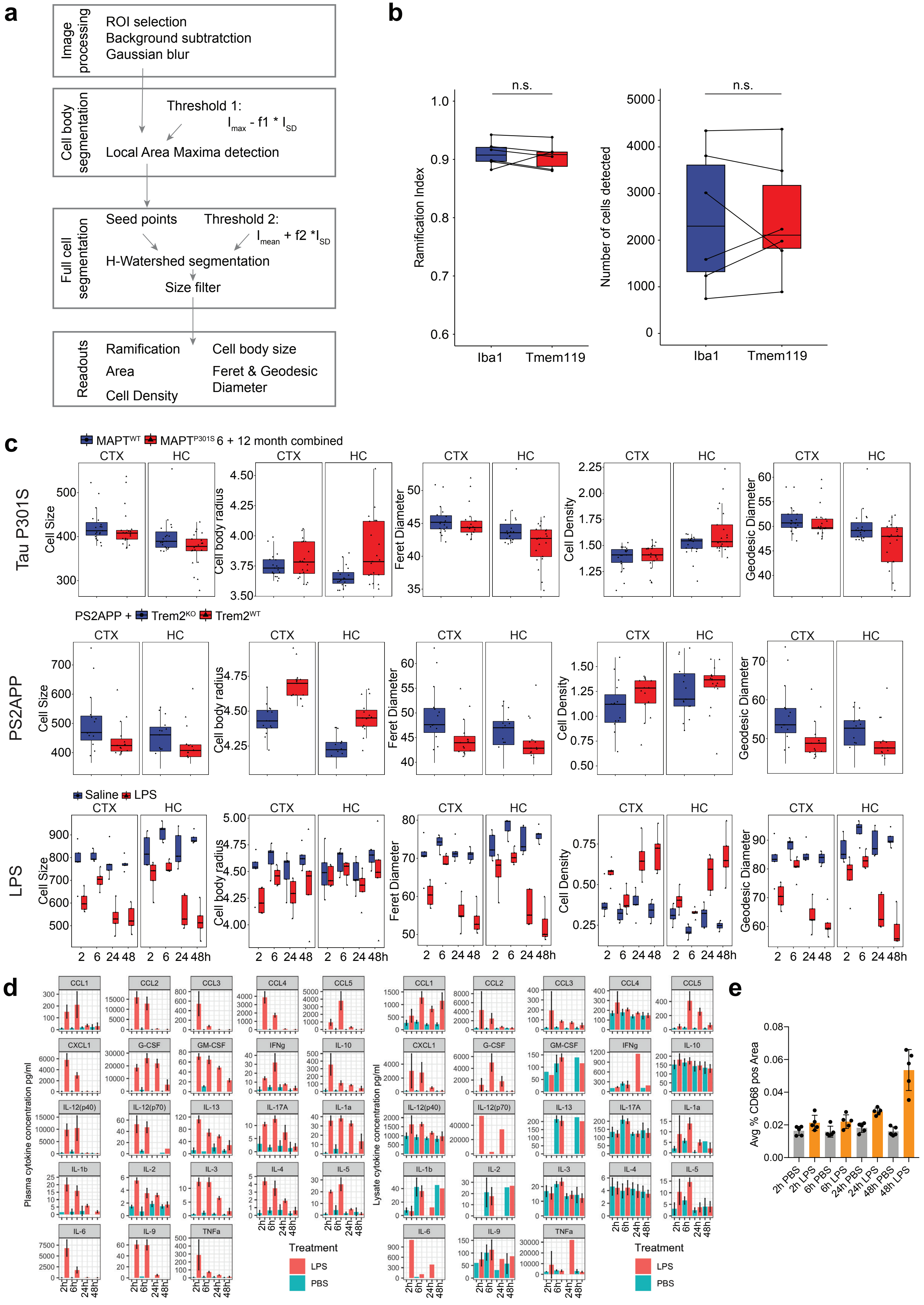
a
Image processing
ROI selection
Background subtratction
Gaussian blur
Cell body segmentation
Threshold 1:
I_{max} - f1 * I_{SD}
Local Area Maxima detection
Full cell segmentation
Seed points
Threshold 2:
I_{mean} + f2 *I_{SD}
H-Watershed segmentation
Size filter
Readouts
Ramification
Area
Cell Density
Cell body size
Feret & Geodesic Diameter
b
n.s.
Ramification Index
Iba1
Tmem119
Number of cells detected
c
MAPT^{WT} MAPT^{P301S} 6 + 12 month combined
Tau P301S
CTX
HC
Cell Size
Cell body radius
Feret Diameter
Cell Density
Geodesic Diameter
PS2APP + Trem2^{KO} Trem2^{WT}
PS2APP
Saline LPS
LPS
2 6 24 48
2 6 24 48h
d
CCL1
CCL2
CCL3
CCL4
CCL5
CXCL1
G-CSF
GM-CSF
IFNg
IL-10
IL-12(p40)
IL-12(p70)
IL-13
IL-17A
IL-1a
IL-1b
IL-2
IL-3
IL-4
IL-5
IL-6
IL-9
TNFa
Plasma cytokine concentration pg/ml
Lysate cytokine concentration pg/ml
2h 6h 24h 48h
Treatment
LPS
PBS
e
Avg % CD68 pos Area
2h PBS
2h LPS
6h PBS
6h LPS
24h PBS
24h LPS
48h PBS
48h LPS

Supplementary Figure 1 Extended characterization of microglial morphology *in-situ*

a) Stepwise workflow of single cell segmentation shown in Figure 1a.

b) Comparison of single cell segmentation results from microglia staining with Iba1 and Tmem119 antibodies of adjacent tissues slices. Datapoints are averages per animal linked by animal. n = 6 animals.

c) Additional morphological descriptors for microglia in neurodegenerative models shown in Figure 1. All datapoints are averaged values per animal.

d) Quantification of cytokines in blood plasma and cerebellar lysate of mice injected *i.p.* with LPS or vehicle at timepoints indicated. Bars indicate mean ± SD. Measurements out of detection range are indicated by n.d. n = 3 replicates.

e) Quantification of CD68 signal in hemibrain in mice injected *i.p.* with LPS or vehicle at timepoints indicated. Bars indicate mean ± SE, n = 5 animals per group.

Statistical significance was calculated paired, two-sided t-tests in b. Boxplots show all datapoints, median, 25th and 75th percentile, whiskers are 1.5*IQR. Source data are provided as a Source Data file.

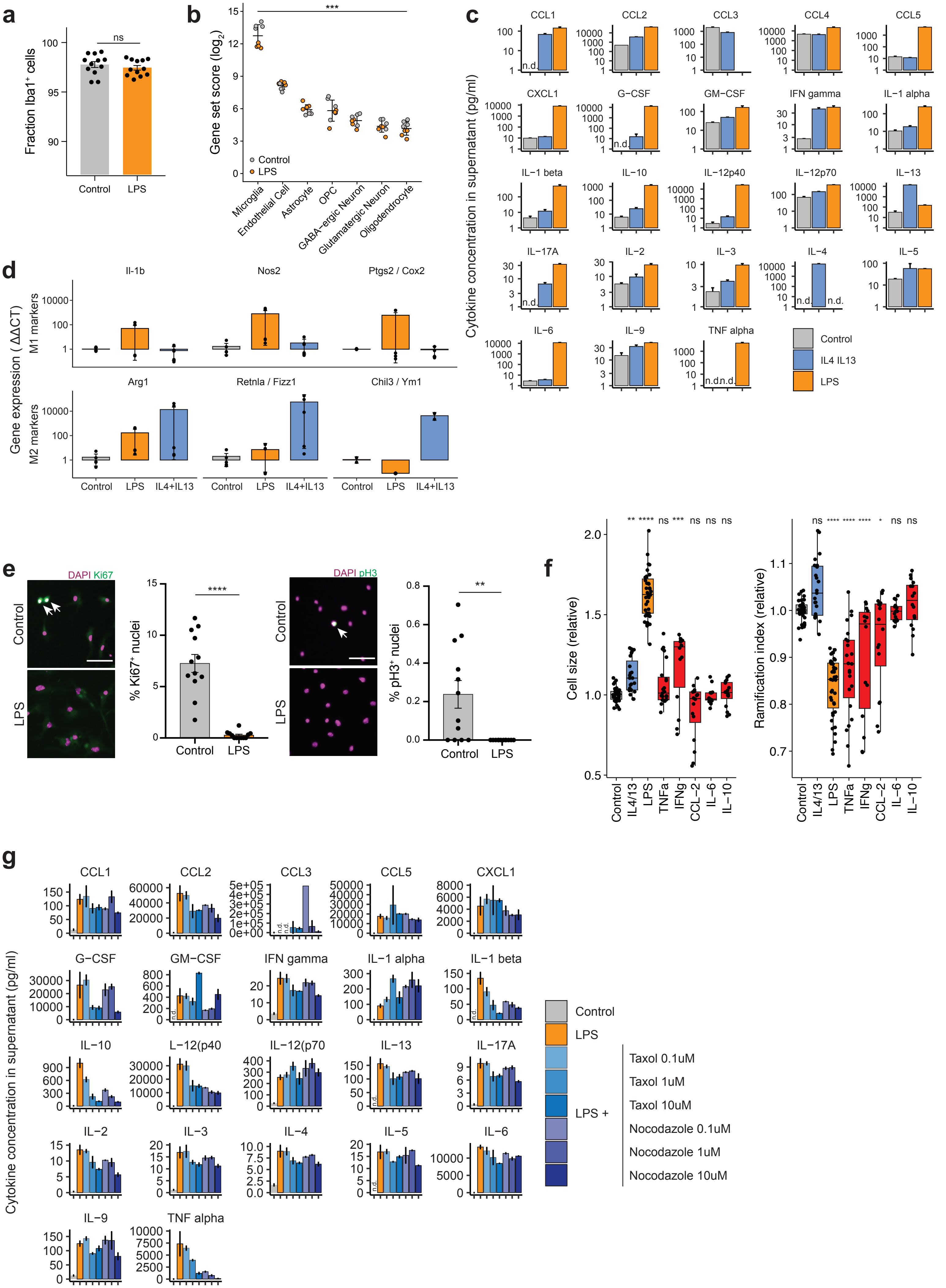

a
Fraction Iba1+ cells
ns
Control
LPS
b
Gene set score (log2)
***
Control
LPS
Microglia
Endothelial Cell
Astrocyte
OPC
GABA-ergic Neuron
Glutamatergic Neuron
Oligodendrocyte
c
Cytokine concentration in supernatant (pg/ml)
CCL1
CCL2
CCL3
CCL4
CCL5
CXCL1
G-CSF
GM-CSF
IFN gamma
IL-1 alpha
IL-1 beta
IL-10
IL-12p40
IL-12p70
IL-13
IL-17A
IL-2
IL-3
IL-4
IL-5
IL-6
IL-9
TNF alpha
n.d.
Control
IL4 IL13
LPS
d
Gene expression (ΔΔCT)
M1 markers
M2 markers
Il-1b
Nos2
Ptgs2 / Cox2
Arg1
Retnla / Fizz1
Chil3 / Ym1
Control
LPS
IL4+IL13
e
DAPI Ki67
DAPI pH3
Control
LPS
% Ki67+ nuclei
% pH3+ nuclei
****
**
f
Cell size (relative)
Ramification index (relative)
** **** ns *** ns ns ns
ns **** **** **** * ns ns
Control
IL4/13
LPS
TNFa
IFNg
CCL-2
IL-6
IL-10
g
Cytokine concentration in supernatant (pg/ml)
CCL1
CCL2
CCL3
CCL5
CXCL1
G-CSF
GM-CSF
IFN gamma
IL-1 alpha
IL-1 beta
IL-10
L-12(p40
IL-12(p70
IL-13
IL-17A
IL-2
IL-3
IL-4
IL-5
IL-6
IL-9
TNF alpha
n.d.
Control
LPS
LPS +
Taxol 0.1uM
Taxol 1uM
Taxol 10uM
Nocodazole 0.1uM
Nocodazole 1uM
Nocodazole 10uM

Supplementary Figure 2 Microglial polarization *in-vitro* and response to microtubule poisons.

a) Quantification of fraction of Iba-1 positive cells in primary microglial cultures after shake-off, n = 12 regions from 2 independent cultures.

b) Gene set score analysis of bulk RNA sequencing of primary microglia and microglia treated with LPS for 24h. n = 4 independent cultures each.

c) Quantification of cytokines secreted into the supernatant of microglial cultures over 24 h after treatment with LPS or IL4 & IL13. Measurements out of detection range are indicated by n.d. n = 3 replicates.

d) Quantification of pro- and anti-inflammatory marker gene expression in microglia treated with LPS or IL-4 & IL-13 for 24 h. Bars indicate mean ± SE of n = 6 measurements from 2 independent cultures.

e) Immunostaining and quantification of Ki-67 and phosphorylated Histone H3-positive cells in microglia treated with LPS, n = 12 regions from 2 independent cultures.

f) Quantification of cell size and ramification index of microglia treated with LPS and indicated cytokines for 24 h relative to control cells. n = 16-39 wells wells from 6 independent cultures.

g) Quantification of cytokines secreted into the supernatant of microglial cultures over 24 h after treatment with LPS and Taxol or Nocodazole at indicated concentrations. Measurements out of detection range are indicated by n.d. n = 3 replicates.

Scale bars are 50µm in e. Statistical significance was calculated with paired, two-sided t-tests in a, e, Kruskal-Wallis and pairwise Wilcox test in b and ANOVA with Tukey HSD in f. Boxplots show all datapoints, median, 25th and 75th percentile, whiskers are 1.5*IQR. Source data are provided as a Source Data file.

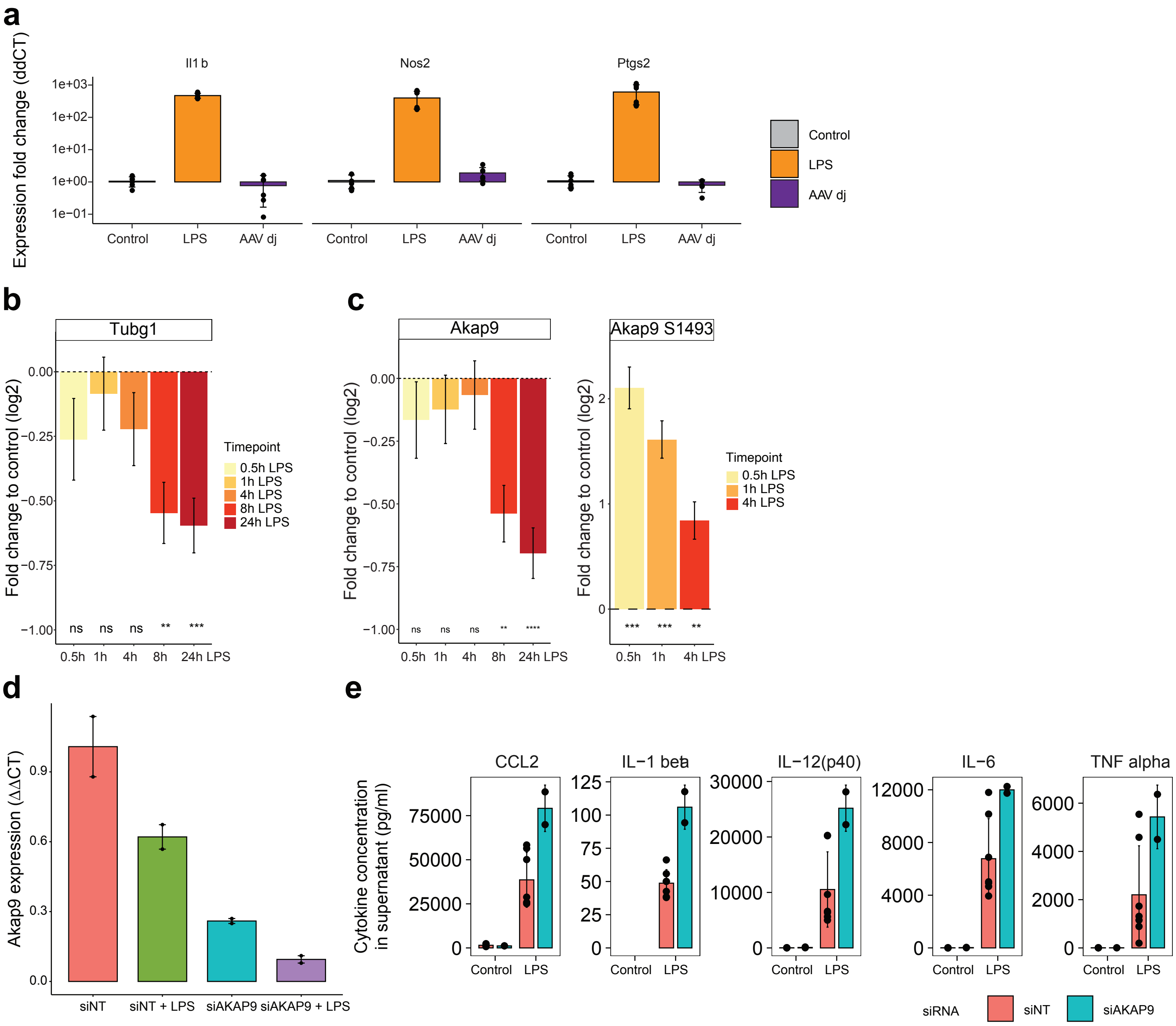
a
Il1 b
Nos2
Ptgs2
Expression fold change (ddCT)
1e+03
1e+02
1e+01
1e+00
1e−01
Control
LPS
AAV dj
Control
LPS
AAV dj
b
Tubg1
Fold change to control (log2)
0.00
−0.25
−0.50
−0.75
−1.00
ns ns ns ** ***
0.5h 1h 4h 8h 24h LPS
Timepoint
0.5h LPS
1h LPS
4h LPS
8h LPS
24h LPS
c
Akap9
Fold change to control (log2)
ns ns ns ** ****
0.5h 1h 4h 8h 24h LPS
Akap9 S1493
*** *** **
0.5h 1h 4h LPS
Timepoint
0.5h LPS
1h LPS
4h LPS
d
Akap9 expression (ΔΔCT)
0.9
0.6
0.3
0.0
siNT
siNT + LPS
siAKAP9
siAKAP9 + LPS
e
Cytokine concentration in supernatant (pg/ml)
CCL2
IL−1 beta
IL−12(p40)
IL−6
TNF alpha
75000
50000
25000
125
100
75
50
25
30000
20000
10000
12000
8000
4000
6000
4000
2000
0
Control
LPS
siRNA
siNT
siAKAP9

Supplementary Figure 3 Extended analysis of microtubule organizing proteins

a) Quantification of inflammatory marker gene expression in microglia treated with AAV to induce protein expression. Bars indicate mean ± SE of n = 3-5 measurements.

b) Quantification of fold changes in protein expression of Tubg1 after LPS treatment compared to control cells as determined by quantitative mass spectrometry.

c) Left: Quantification of fold changes in protein expression of Akap9 after LPS treatment compared to control cells as determined by quantitative mass spectrometry. Right: Quantification of fold changes in Akap9 S1493 phosphorylation after LPS treatment compared to control cells.

d) Quantification of Akap9 expression by qPCR in microglia treated with LPS, siAkap9 or combination of both compared to control cells, n = 2 replicates.

e) Quantification of cytokines secreted into the supernatant of microglial cultures over 24 h after treatment with siNT, siAkap9 and/or LPS. Measurements out of detection range are indicated by n.d. n = 2-5 replicates.

Bars indicate mean ± SE in a, c, d, e and mean ± SD in b. Statistical significance was calculated with ANOVA and Tukey HSD in b, c. Source data are provided as a Source Data file.

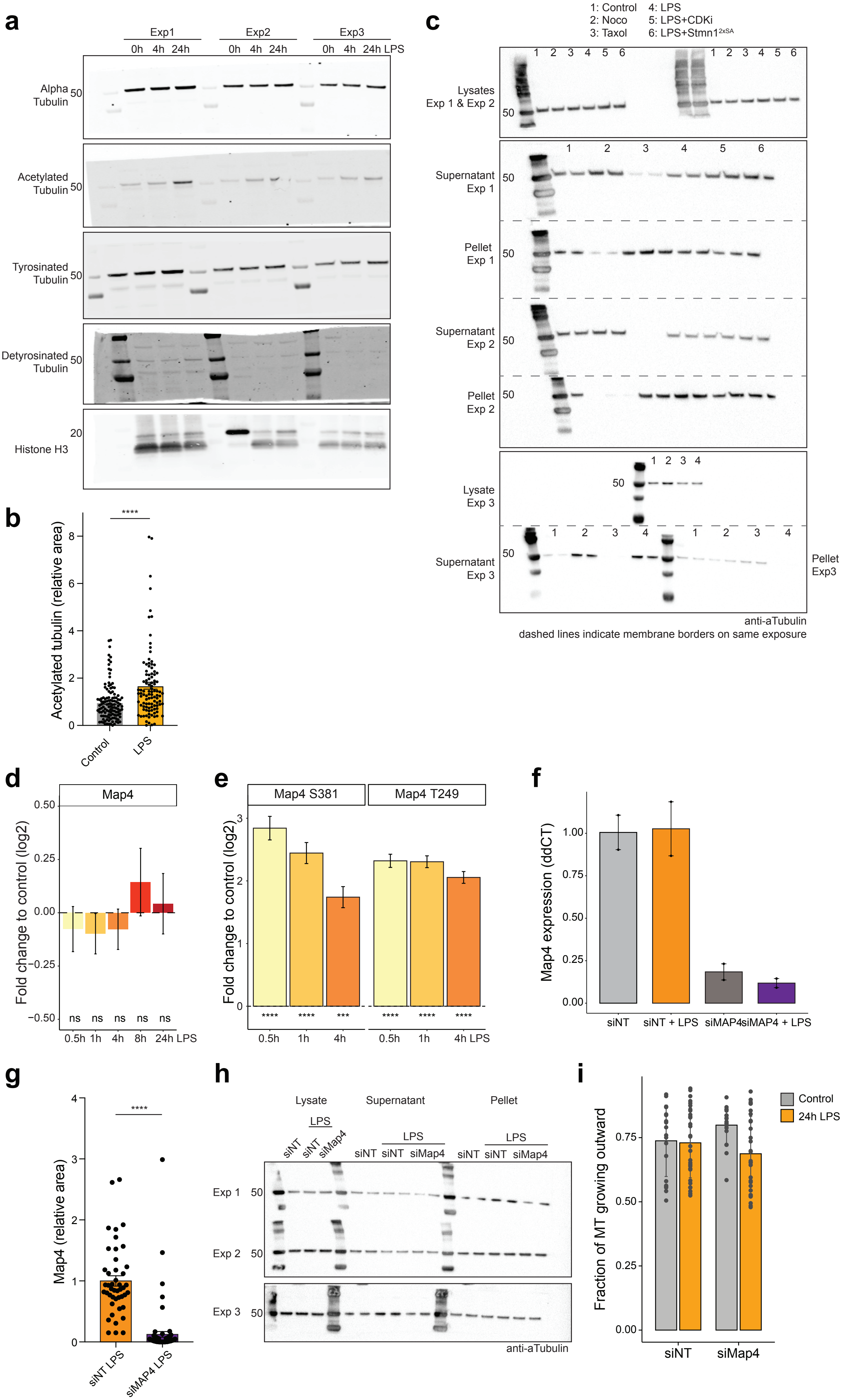
a
Exp1
Exp2
Exp3
0h 4h 24h
0h 4h 24h
0h 4h 24h LPS
Alpha Tubulin 50
Acetylated Tubulin 50
Tyrosinated Tubulin 50
Detyrosinated Tubulin 50
Histone H3 20
b
Acetylated tubulin (relative area)
****
Control
LPS
c
1: Control
2: Noco
3: Taxol
4: LPS
5: LPS+CDKi
6: LPS+Stmn1 2xSA
Lysates Exp 1 & Exp 2
Supernatant Exp 1
Pellet Exp 1
Supernatant Exp 2
Pellet Exp 2
Lysate Exp 3
Supernatant Exp 3
Pellet Exp3
anti-aTubulin
dashed lines indicate membrane borders on same exposure
d
Map4
Fold change to control (log2)
ns ns ns ns ns
0.5h 1h 4h 8h 24h LPS
e
Map4 S381
Map4 T249
Fold change to control (log2)
**** **** ***
**** **** ****
0.5h 1h 4h
0.5h 1h 4h LPS
f
Map4 expression (ddCT)
siNT
siNT + LPS
siMAP4
siMAP4 + LPS
g
Map4 (relative area)
****
siNT LPS
siMAP4 LPS
h
Lysate
Supernatant
Pellet
LPS
siNT siNT siMap4
siNT siNT siMap4
Exp 1 50
Exp 2 50
Exp 3 50
anti-aTubulin
i
Fraction of MT growing outward
Control
24h LPS
siNT
siMap4

Supplementary Figure 4 Extended analysis of Stmn1 and Map4 pathways

a) Full immunoblot panels for microtubule PTM analysis in Figure 3a. Membranes were imaged with Licor Oddysey.

b) Quantification of acetylated tubulin fluorescent signal in Figure 5b. n = 102-120 cells from 4 independent cultures.

c) Full immunoblot panels for microtubule polymerization spin-down assay in Figure 3e. Membranes were imaged with ECL on Biorad Geldoc.

d) Quantification of fold changes in protein levels of Map4 after LPS treatment as determined by quantitative mass spectrometry.

e) Quantification of fold changes in phosphorylation of Map4 S381 and T249, both located in the uncharacterized N-terminus of Map4, after LPS treatment compared to control cells as determined by quantitative mass spectrometry.

f) Quantification of fold changes in expression of Map4 after treatment with LPS and siMap4.

g) Quantification of Map4 fluorescent signal in microglia treated with LPS and siMap4, n = 45-76 cells from 2 independent cultures.

h) Quantification of MT+TIP growth direction for microglia treated with siMap4 and LPS as indicated. n = 25-31 cells per group from 4 independent cultures

i) Full immunoblot panels for microtubule polymerization spin-down assay in Figure 3j. Membranes were imaged with ECL on Biorad Geldoc.

Bars indicate mean ± SE in d, e, fand mean ± SD in b, g, h. Statistical significance was calculated with ANOVA and Tukey HSD in d, e, h and unpaired, two-sided t-tests in b, g. Source data are provided as a Source Data file.

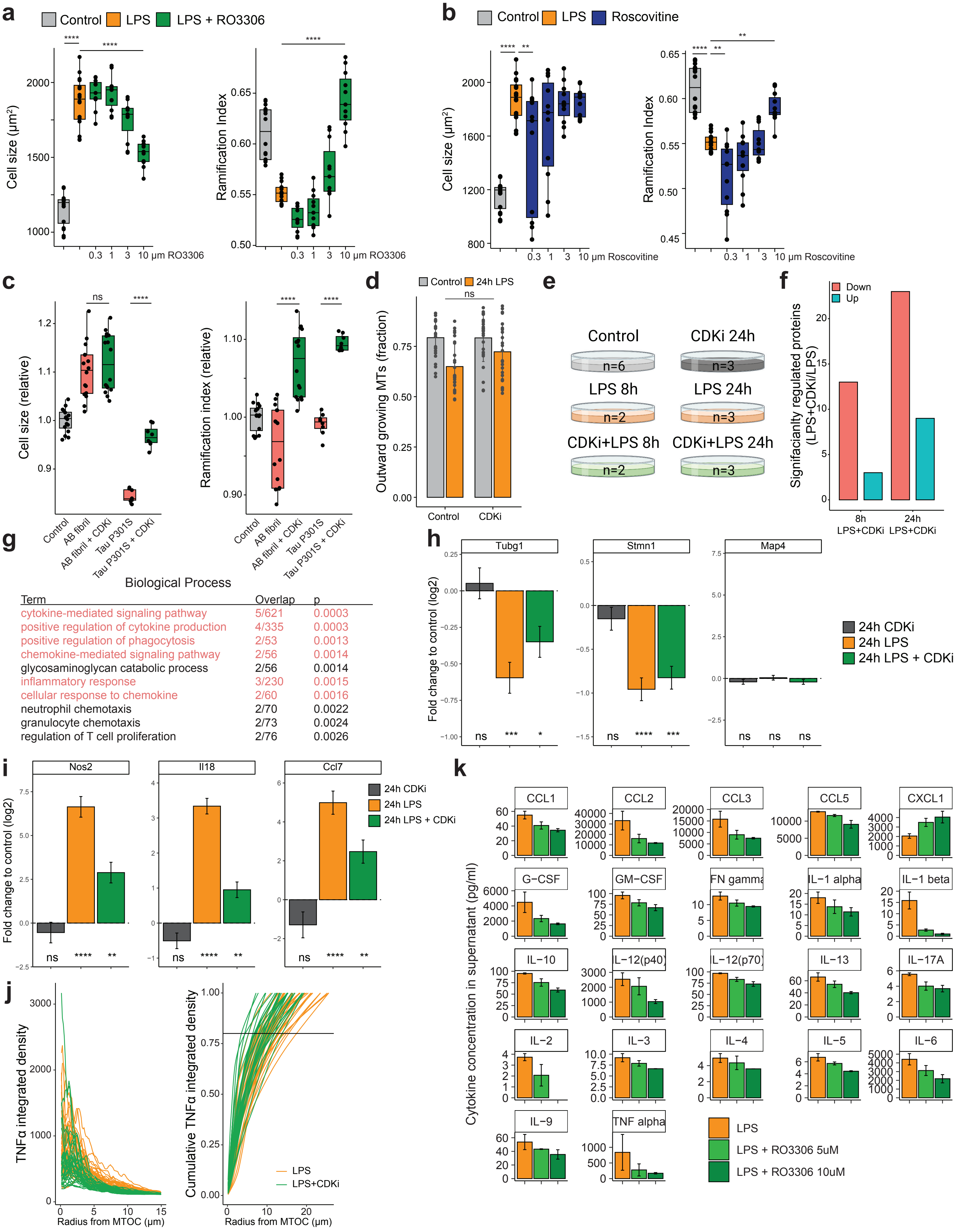
a
Control
LPS
LPS + RO3306
Cell size (µm²)
Ramification Index
0.3 1 3 10 µm RO3306
b
Control
LPS
Roscovitine
0.3 1 3 10 µm Roscovitine
c
Cell size (relative)
Ramification index (relative)
Control
AB fibril
AB fibril + CDKi
Tau P301S
Tau P301S + CDKi
d
Control
24h LPS
Outward growing MTs (fraction)
Control
CDKi
e
Control
CDKi 24h
LPS 8h
LPS 24h
CDKi+LPS 8h
CDKi+LPS 24h
n=6
n=3
n=2
n=3
n=2
n=3
f
Down
Up
Signficianlty regulated proteins (LPS+CDKi/LPS)
8h LPS+CDKi
24h LPS+CDKi
g
Biological Process
Term | Overlap | p
cytokine-mediated signaling pathway | 5/621 | 0.0003
positive regulation of cytokine production | 4/335 | 0.0003
positive regulation of phagocytosis | 2/53 | 0.0013
chemokine-mediated signaling pathway | 2/56 | 0.0014
glycosaminoglycan catabolic process | 2/56 | 0.0014
inflammatory response | 3/230 | 0.0015
cellular response to chemokine | 2/60 | 0.0016
neutrophil chemotaxis | 2/70 | 0.0022
granulocyte chemotaxis | 2/73 | 0.0024
regulation of T cell proliferation | 2/76 | 0.0026
h
Tubg1
Stmn1
Map4
Fold change to control (log2)
24h CDKi
24h LPS
24h LPS + CDKi
i
Nos2
Il18
Ccl7
Fold change to control (log2)
24h CDKi
24h LPS
24h LPS + CDKi
j
TNFα integrated density
Cumulative TNFα integrated density
Radius from MTOC (µm)
LPS
LPS+CDKi
k
CCL1
CCL2
CCL3
CCL5
CXCL1
G-CSF
GM-CSF
FN gamma
IL-1 alpha
IL-1 beta
IL-10
IL-12(p40)
IL-12(p70)
IL-13
IL-17A
IL-2
IL-3
IL-4
IL-5
IL-6
IL-9
TNF alpha
Cytokine concentration in supernatant (pg/ml)
LPS
LPS + RO3306 5uM
LPS + RO3306 10uM

Supplementary Figure 5 Extended analysis of Cdk1 pathway

a) Quantification of cell size and ramification index of microglia treated with LPS and increasing doses of RO3306. n = 11-16 wells per condition from 3 independent cultures.

b) Quantification of cell size and ramification index of microglia treated with LPS and increasing doses of roscovitine. n = 11-16 wells per condition from 3 independent cultures.

c) Quantification of cell size and ramification index of microglia treated with indicated stimulations and RO3306 for 24 h relative to controls. n = 8-16 wells per condition from 4 independent cultures.

d) Quantification of MT+TIP growth direction for microglia treated with RO3306 and LPS. n = 25-26 wells per condition from 5 independent cultures.

e) Experimental conditions for proteomic analysis: Primary microglia cultures were distributed over the conditions indicated, before lysis, TMT-multiplexing and proteomic analysis.

f) Quantification of significantly up- and down-regulated proteins in proteomic analysis of microglia treated with LPS and RO3306 compared to LPS alone. For full table see Supplementary Data 1.

g) Gene ontology analysis of biological processes enriched in proteins down-regulated after 8h of LPS and RO3306 treatment compared to LPS alone. Terms associated with inflammation are highlighted in red. For full table see Supplementary Data 2.

h) Quantification of the fold change in protein levels of Tubg1, Stmn1 and Map4 in microglia treated with RO3306 alone (grey), LPS alone (yellow) and LPS and RO3306 (green) for 24 h as determined by quantitative mass spectrometry.

i) Quantification of the fold change in protein levels of Nos2, IL-18 and Ccl7 in microglia treated with RO3306 alone (grey), LPS alone (yellow) and LPS and RO3306 (green) for 24h as determined by quantitative mass spectrometry.

j) Quantification of TNFa immunostaining distribution in primary microglia summarized in Figure 6j. Left: TNFa signal as a function of cell radius centered on the MTOC. Right: Cumulative TNFa signal as a function of cell radius centered on the MTOC. Lines indicate individual cells.

k) Quantification of chemokine and cytokine secretion from microglia treated with RO3306 and LPS as indicated into their supernatant for 24 h.

Bars indicate mean ± SD in a, b, c, d, k and mean ± SE in h, i. Boxplots show all datapoints, median, 25th and 75th percentile, whiskers are 1.5*IQR. Statistical significance was calculated with ANOVA and Tukey HSD in a, b, c, d, h, j. Source data are provided as a Source Data file.
