# Supplementary material for: Polarized microtubule remodeling transforms the morphology of reactive microglia and drives cytokine release
Source: Nat Commun. 2023 Oct 9;14:6322. doi: 10.1038/s41467-023-41891-6 (PMC10562429; doi:10.1038/s41467-023-41891-6)
Supplement: Supplementary file 2 — Reporting Summary [file 41467_2023_41891_MOESM2_ESM.pdf]

nature portfolio

Corresponding author(s): Casper Hoogenraad

Last updated by author(s): Aug 27, 2023

Reporting Summary

Nature Portfolio wishes to improve the reproducibility of the work that we publish. This form provides structure for consistency and transparency in reporting. For further information on Nature Portfolio policies, see our Editorial Policies and the Editorial Policy Checklist.

Statistics

For all statistical analyses, confirm that the following items are present in the figure legend, table legend, main text, or Methods section.

n/a	Confirmed	
☐	☒	The exact sample size (*n*) for each experimental group/condition, given as a discrete number and unit of measurement
☐	☒	A statement on whether measurements were taken from distinct samples or whether the same sample was measured repeatedly
☐	☒	The statistical test(s) used AND whether they are one- or two-sided<br>*Only common tests should be described solely by name; describe more complex techniques in the Methods section.*
☐	☒	A description of all covariates tested
☐	☒	A description of any assumptions or corrections, such as tests of normality and adjustment for multiple comparisons
☐	☒	A full description of the statistical parameters including central tendency (e.g. means) or other basic estimates (e.g. regression coefficient) AND variation (e.g. standard deviation) or associated estimates of uncertainty (e.g. confidence intervals)
☐	☒	For null hypothesis testing, the test statistic (e.g. *F*, *t*, *r*) with confidence intervals, effect sizes, degrees of freedom and *P* value noted<br>*Give P values as exact values whenever suitable.*
☒	☐	For Bayesian analysis, information on the choice of priors and Markov chain Monte Carlo settings
☒	☐	For hierarchical and complex designs, identification of the appropriate level for tests and full reporting of outcomes
☐	☒	Estimates of effect sizes (e.g. Cohen's *d*, Pearson's *r*), indicating how they were calculated

Our web collection on statistics for biologists contains articles on many of the points above.

Software and code

Policy information about availability of computer code

Data collection	Software used to acquire data is detailed in the respective materials and methods sections. Software used includes Slidebook 6 (Intelligent Imaging Innovations), Prairie View Imaging (Bruker), QuantStudio 7 Flex (Thermo Fisher), GelDoc XR+ (BioRad), Odyssey (Licor)
Data analysis	Data tables were tidied, filtered and summarized in R (version 4.2.3) in RStudio (version 2023.03.0 Build 386) using tidyverse 2.0.0 and statistically evaluated with rstatix 0.7.2 using two-sided t-test or one-way ANOVA and Tukey's HSD as indicated. Plots were generated using ggplot2 3.4.2 or ggpubr 0.6.0 with significance intervals at **** < 1e-04 < *** < 0.001 < ** < 0.01 < * < 0.05 < ns. Alternatively, we used Graph Pad Prism 9 for plotting and statistical analysis with two-sided t-tests and one-way ANOVA with Dunnett's or Tukey's multiple comparison tests as indicated. All source data for graphs and their statistical analyses are reported in the Source Data.<br>The HTSeqGenie R package (v4.30, doi:10.18129/B9.bioc.HTSeqGenie) was used to process RNA-seq reads, including filtering, alignment and feature counting.<br>Assignment of MS/MS spectra was performed using the MASCOT search algorithm to search against all entries for Mus musculus (house mouse) in UniProt (downloaded June 2016 for LPS+CDKi or August 2017 for LPS experiments). Quantitative MS values were extracted and corrected for isotopic impurities using Mojave 70. Additionally, quantitative events with a precursor purity < 0.5 or 0.7 (± 0.25 Da). The R package MSstatsTMT v.1.6.3 (LPS) or v1.6.6 (LPS+CDKi) was used to preprocess PSM-level quantification before statistical analysis, to have protein quantification and to perform differential abundance analysis. MSstatsTMT estimated log2(fold change) and the standard error by linear mixed effect model for each protein. Resulting protein lists were submitted to EnrichR GO analysis through enrichR R package (v3.0, https://maayanlab.cloud/Enrichr/) .Overlays for volcano plots were generated by highlighting proteins associated with GO terms indicated as found on AmiGO 72 (http://amigo.geneontology.org/amigo).<br>MT+TIP tracks were analysed with TrackMate 7.6<br>Volumetric analysis of microglial morphology from 2P microscopy was performed by surface segmentation in Imaris 10.1 (Oxford Instruments).

Image analysis developed for this paper is based on publicly available functions described in Material & Methods and scripts are available on github [https://github.com/maxadrian/mg-segmentation/].

For manuscripts utilizing custom algorithms or software that are central to the research but not yet described in published literature, software must be made available to editors and reviewers. We strongly encourage code deposition in a community repository (e.g. GitHub). See the Nature Portfolio guidelines for submitting code & software for further information.

Data

Policy information about availability of data

All manuscripts must include a data availability statement. This statement should provide the following information, where applicable:

- Accession codes, unique identifiers, or web links for publicly available datasets
- A description of any restrictions on data availability
- For clinical datasets or third party data, please ensure that the statement adheres to our policy

All data used in plots throughout the paper and their statistical analyses are available in the Source Data file.
The quantitative microscopy, Luminex, qPCR and immunoblot data generated in this study are provided in the Source Data file.
The raw proteomic data generated in this study have been deposited in the MassIVE repository under accession code MSV000090254 [doi:10.25345/C5TT4FZ0P].
The processed proteomics data are available in the Supplementary Information.
The RNA sequencing data generated in this study have been deposited in the NCBI GEO database under accession code GSE238210 [https://www.ncbi.nlm.nih.gov/geo/query/acc.cgi?acc=GSE238210].

Research involving human participants, their data, or biological material

Policy information about studies with human participants or human data. See also policy information about sex, gender (identity/presentation), and sexual orientation and race, ethnicity and racism.

Reporting on sex and gender	N/A
Reporting on race, ethnicity, or other socially relevant groupings	N/A
Population characteristics	N/A
Recruitment	N/A
Ethics oversight	N/A

Note that full information on the approval of the study protocol must also be provided in the manuscript.

Field-specific reporting

Please select the one below that is the best fit for your research. If you are not sure, read the appropriate sections before making your selection.

☒ Life sciences ☐ Behavioural & social sciences ☐ Ecological, evolutionary & environmental sciences

For a reference copy of the document with all sections, see nature.com/documents/nr-reporting-summary-flat.pdf

Life sciences study design

All studies must disclose on these points even when the disclosure is negative.

Sample size	No sample size calculations were performed prior to the studies. Animal studies were kept to a minimum number of animals for ethical reasons, typically 5-15 animals per group. Experiments with primary microglia were repeated with cells from independent cultures as indicated in the figure legends (n=2-5)
Data exclusions	We excluded 4 mice from the acute slices experiment in Figure 7 and state so in the Methods section. The analysis of microglial responses in acute slices is complicated by the tissue injuring through slicing per se. We therefor excluded mice that showed strong morphological activation of microglia at the target depth in control conditions that did not show an increased morphological phenotype after LPS stimulation.
Replication	All studies have been repeated in independent cultures of primary cells or in multiple animals (n =2 -5) , as indicated in figure legends. In addition, cell morphology assays, qPCR and Luminex assays were measured in technical replicates that were averaged into datapoints shown, as indicated in figure legends. No replicates have been excluded.
Randomization	Mice were randomized and kept with randomized littermates during the study and identified by tail marks.
Blinding	Animal handlers were blinded to injections. Investigators where blinded while drawing ROIs or manually analyzing images. The majority of data analysis was scripted and same parameters applied to all samples.

Reporting for specific materials, systems and methods

We require information from authors about some types of materials, experimental systems and methods used in many studies. Here, indicate whether each material, system or method listed is relevant to your study. If you are not sure if a list item applies to your research, read the appropriate section before selecting a response.

Materials & experimental systems

n/a	Involved in the study
☐	☒ Antibodies
☒	☐ Eukaryotic cell lines
☒	☐ Palaeontology and archaeology
☐	☒ Animals and other organisms
☒	☐ Clinical data
☒	☐ Dual use research of concern
☒	☐ Plants

Methods

n/a	Involved in the study
☒	☐ ChIP-seq
☒	☐ Flow cytometry
☒	☐ MRI-based neuroimaging

Antibodies

Antibodies used

We used the following primary antibodies in this study:
Target Dilution Animal Fixation Supplier Catalog# Lot No
Camsap1l1 (Camsap2) 500 rabbit PFA Proteintech 17880-1-AP N/A
EB1 1000 mouse MeOH BD 610535 6078948
GM130 500 rabbit MeOH Abcam ab52649 N/A
Iba1 500 rabbit PFA Wako 019-19741 LEJ1842
Pericentrin 5000 rabbit PFA Biolegend 923701 B215567
Tubulin alpha 1000 mouse MeOH/PFA Sigma T6199 029M4842V
Tubulin acetylated 500 mouse PFA Millipore T7451 206322
Map4 500 rabbit MeOH Abcam ab245578 GR3259936-3
Histone H3 (pS28) 1000 rat PFA Abcam ab10543 N/A
Ki-67 500 mouse PFA BD 550609 N/A
Histone H3 500 rabbit WB Cell Signaling 4499S 9
Tubulin acetylated 500 rabbit PFA/WB Cell Signaling 5335S 5
Tubulin detyrosinated 1000 rabbit WB Sigma AB3201 N/A

For secondary fluorescent antibodies, we used goat anti-mouse IgG Alexa Fluor Plus 488 (A32723, LOT VA288487) or Alexa Fluor Plus 647 (A32728, LOT UK290265), Alexa Fluor 568 goat anti-mouse IgG (A11031 LOT 2124366), goat anti-rabbit IgG Alexa Fluor Plus 488 (A32731, LOT UK290266) or Alexa Fluor Plus 647 (A32733, LOT UL291628) and Alexa Fluor 568 goat anti-rabbit IgG (A11036, LOT 2155282, all Invitrogen), all at 1:1000 dilution in blocking solution.
For immunoblots we used the following secondary antibodies: Anti-mouse IgG HRP-linked (7076, LOT 34) and anti-rabbit IgG HRP linked (7074, LOT 29, both Cell signaling) or IRDye800CW donkey anti-mouse (926-32212 LOT D21109-15) and IRDye680RD goat-anti-rabbit (926-68071, LOT D00819-05, both Licor), all at 1:1000 dilution.
Iba1 IHC Stain:
Primary Antibody: Iba1 IHC Secondary Antibody: Anti-Rabbit Biotinylated
Source: Abcam Source: Vector
Catalog #: ab178846 Catalog #: BA-1000
Host: Rabbit Host: Goat
Dilutions: 75,000 Dilution: 1:1,000
Chromagen: Ni(II)-DAB
Color: Black
CD68 IHC Stain:
Primary Antibody: CD68 IHC Secondary Antibody: Anti-Rabbit Biotinylated
Source: Rockland Source: Vector
Catalog #: 600-401-R10 Catalog #: BA-1000
Host: Rabbit Host: Goat
Dilutions: 50,000 Dilution: 1:1,000
Chromagen: Ni(II)-DAB
Color: Black

Validation

No antibody validation in addition to the vendors' statements have been performed in this study.
ab178846 has been referenced in 285 publications. https://www.abcam.com/products/primary-antibodies/iba1-antibody-epr16588-ab178846.html
600-401-R10 has been referenced in 1 publication. https://www.rockland.com/categories/primary-antibodies/cd68-antibody-600-401-R10/
17880-1-AP has been referenced in 52 publications. https://www.ptglab.com/products/CAMSAP1L1-Antibody-17880-1-AP.htm
610535 has been referenced in 5 publications. https://www.bdbiosciences.com/en-us/products/reagents/microscopy-imaging-reagents/immunofluorescence-reagents/purified-mouse-anti-eb1.610535

ab52649 has been referenced in 250 publications. https://www.abcam.com/products/primary-antibodies/gm130-antibody-ep892y-cis-golgi-marker-ab52649.html
019-19741 has been referenced in 3838 publications. https://labchem-wako.fujifilm.com/us/product/detail/W01W0101-1974.html
923701 has been referenced in 15 publications. https://www.citeab.com/antibodies/2864082-923701-anti-pericentrin-antibody
T6199 has been referenced in 2119 publications. https://www.sigmaaldrich.com/US/en/product/sigma/t6199
T7451 has been referenced in 1039 publications. https://www.sigmaaldrich.com/US/en/product/sigma/t7451
ab245578 has been referenced in 3 publications. https://www.abcam.com/products/primary-antibodies/map4-antibody-ab245578.html
ab10543 has been referenced in 106 publications. https://www.abcam.com/products/primary-antibodies/histone-h3-phospho-s28-antibody-hta28-ab10543.html
550609 has been referenced in 14 publications. https://www.bdbiosciences.com/en-us/products/reagents/flow-cytometry-reagents/research-reagents/single-color-antibodies-ruo/purified-mouse-anti-ki-67.550609
4499S has been referenced in 1504 publications. https://www.cellsignal.com/products/primary-antibodies/histone-h3-d1h2-xp-rabbit-mab/4499?_requestid=627295
5335S has been referenced in 330 publications. https://www.cellsignal.com/products/primary-antibodies/acetyl-a-tubulin-lys40-d20g3-xp-rabbit-mab/5335
AB3201 has been referenced in 152 publications. https://www.emdmillipore.com/US/en/product/Anti-Tubulin-Antibody-Detyrosinated,MM_NF-AB3201

Animals and other research organisms

Policy information about studies involving animals; ARRIVE guidelines recommended for reporting animal research, and Sex and Gender in Research

Laboratory animals

Animals were housed in specific pathogen-free conditions with 14 h light/10 h dark/day and maintained on regular chow diets and tap water ad libitum.
Wildtype C57BL/6N pups were obtained from Charles River, Hollister (CA). C57BL6/J mice were obtained from Jackson Laboratories, Sacramento (CA). Male and female TauP301S mice aged 6 and 12 months (REF23,24), female PS2APP x Trem2KO mice at 6 months (REF20,60) and male and female Cx3CR1wt/GFP mice (REF39) were bred at Genentech.
For the LPS time course study, ~10-week-old C57BL6/J male mice were injected with 1 mg/kg LPS from Salmonella enterica (L6143, Sigma-Aldrich, St. Louis, MO) i.p. or vehicle control (PBS). Application volume was 10ml/kg. Animals were monitored and weighted beginning on the day before treatment at timepoints indicated.
All animals were anaesthetized with 2.5 % Avertin (2,2,2-tribromoethanol (Sigma-Aldrich); ~0.5 ml/25 g body weight) and transcardially perfused with cold PBS.
Primary murine microglia were harvested from mixed glial cultures by shake-off from triturated neonatal P2-P3 mouse brains, that were collected after decapitation.

Wild animals

No wild animals were used in this study.

Reporting on sex

Male and female TauP301S mice aged 6 and 12 months.
Female PS2APP x Trem2KO mice at 6 months.
Three-month-old male and female CX3CR1wt/GFP mice (n = 9) were used for acute slice preparation.
We did not observe or expect sex-dependent effects, see Source Data for Fig1.
Pups used for microglial cultures were not sexed and pooled during culturing.

Field-collected samples

No field-collected samples were used in this study.

Ethics oversight

All animal care and handling procedures were reviewed and approved by the Genentech IACUC and were conducted in full compliance with IACUC policies and the Institute for Lab Animals' guidelines for the humane care and use of laboratory animals.

Note that full information on the approval of the study protocol must also be provided in the manuscript.
