# Supplementary material for: Polarized microtubule remodeling transforms the morphology of reactive microglia and drives cytokine release
Source: Nat Commun. 2023 Oct 9;14:6322. doi: 10.1038/s41467-023-41891-6 (PMC10562429; doi:10.1038/s41467-023-41891-6)
Supplement: Supplementary file 4 — Description of Additional Supplementary files [file 41467_2023_41891_MOESM4_ESM.pdf]

Description of additional Supplementary files

Supplementary Movie 1
MT+TIP tracking in primary microglial cells Representative MT+TIP imaging in live primary microglia shown in Fig. 4d. Top panels show control cell and bottom panel shows cell treated with LPS for 24 h prior to imaging. Images were taken at 1 fps for 5 min. Left panels show raw images, middle panels show spot detection of MT+TIPs and right panels show tracks generated in TrackMate that were analyzed in Fig. 4e. Time is shown in mm:ss and scale bars are 10μm.

Supplementary Movie 2
MT+TIP imaging after nocodazole washout Representative MT+TIP imaging in live primary microglia immediately after nocodazole washout. Images were taken at 1 fps for 5 min. Left panel shows control cells after washout of 1μM nocodazole applied for 1h. Right panel shows cells that were treated with LPS for 16 h before nocodazole washout assay. Time is shown in mm:ss and scale bars are 10μm.

Supplementary Movie 3
Threedimensional images of microglia in acute brain slices Animation of 3D image stacks shown in Fig. 7b. Panels show representative slices in control (top left), LPS-treated (top right), CDKi-treated (bottom left) and LPS + CDKi-treated (bottom right) slices. Scale bar is 30μm and cell volumes are color coded as indicated between 500 and 5000 μm3.

Supplementary Data 1
Quantitative Proteomic Results Table containing fold-change values and statistical analysis of quantitative proteomics and phospho-proteomics shown throughout this study. Comparisons are LPS vs Control, CDKi vs Control and LPS + CDKi vs LPS. To test two-sided null hypothesis of no changes in abundance, the model-based test statistics were compared to the Student t-test distribution with the degrees of freedom appropriate for each protein and each dataset. The resulting P values were adjusted to control the FDR with the method by Benjamini– Hochberg. For raw proteomic data, see Data Availability.

Supplementary Data 2
Gene Ontology Results Extended Gene Ontology analyses for Fig. 3c, d, h, i and Supplementary Fig. 5g and list of gene names used for highlighting GO terms in volcano plots in Fig. 3e, f, j, k and 6i. P values are derived from Fisher's exact test and adjusted with Benjamini-Hochberg for multiple hypothesis testing.
