# Supplementary material for: Polarized microtubule remodeling transforms the morphology of reactive microglia and drives cytokine release
Source: Nat Commun. 2023 Oct 9;14:6322. doi: 10.1038/s41467-023-41891-6 (PMC10562429; doi:10.1038/s41467-023-41891-6)
Supplement: Supplementary file 10 — Source Data [file 41467_2023_41891_MOESM10_ESM.zip › data/Fig S1/FigS1b_Tmem_t-test.rtf]

	Paired t-testdata:  Ramification by Staint = 0.62466, df = 5, p-value = 0.5596alternative hypothesis: true mean difference is not equal to 095 percent confidence interval: -0.01411408  0.02317559sample estimates:mean difference     0.004530753 	Paired t-testdata:  n by Staint = -0.0056215, df = 5, p-value = 0.9957alternative hypothesis: true mean difference is not equal to 095 percent confidence interval: -763.7895  760.4562sample estimates:mean difference       -1.666667 
