# Supplementary figures and images for: Polarized microtubule remodeling transforms the morphology of reactive microglia and drives cytokine release
Source: Nat Commun. 2023 Oct 9;14:6322. doi: 10.1038/s41467-023-41891-6 (PMC10562429; doi:10.1038/s41467-023-41891-6)

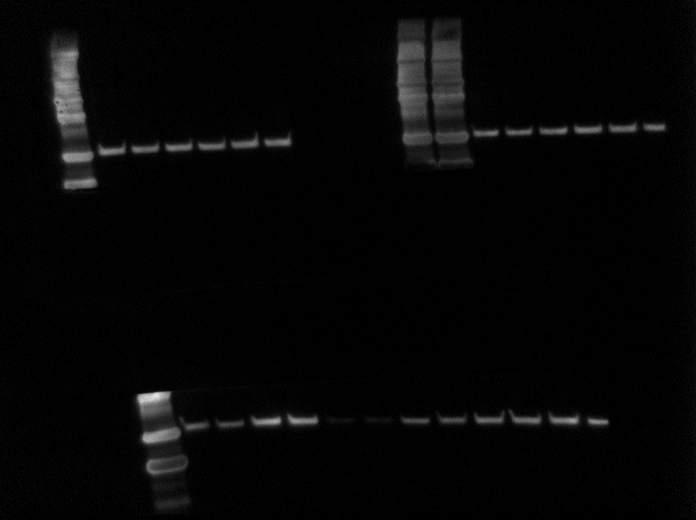

Supplement: Supplementary file 10 — Source Data [file 41467_2023_41891_MOESM10_ESM.zip › data/Fig 5/Fig5e:S4c uncropped blot Exp1&2 lysate.tif]

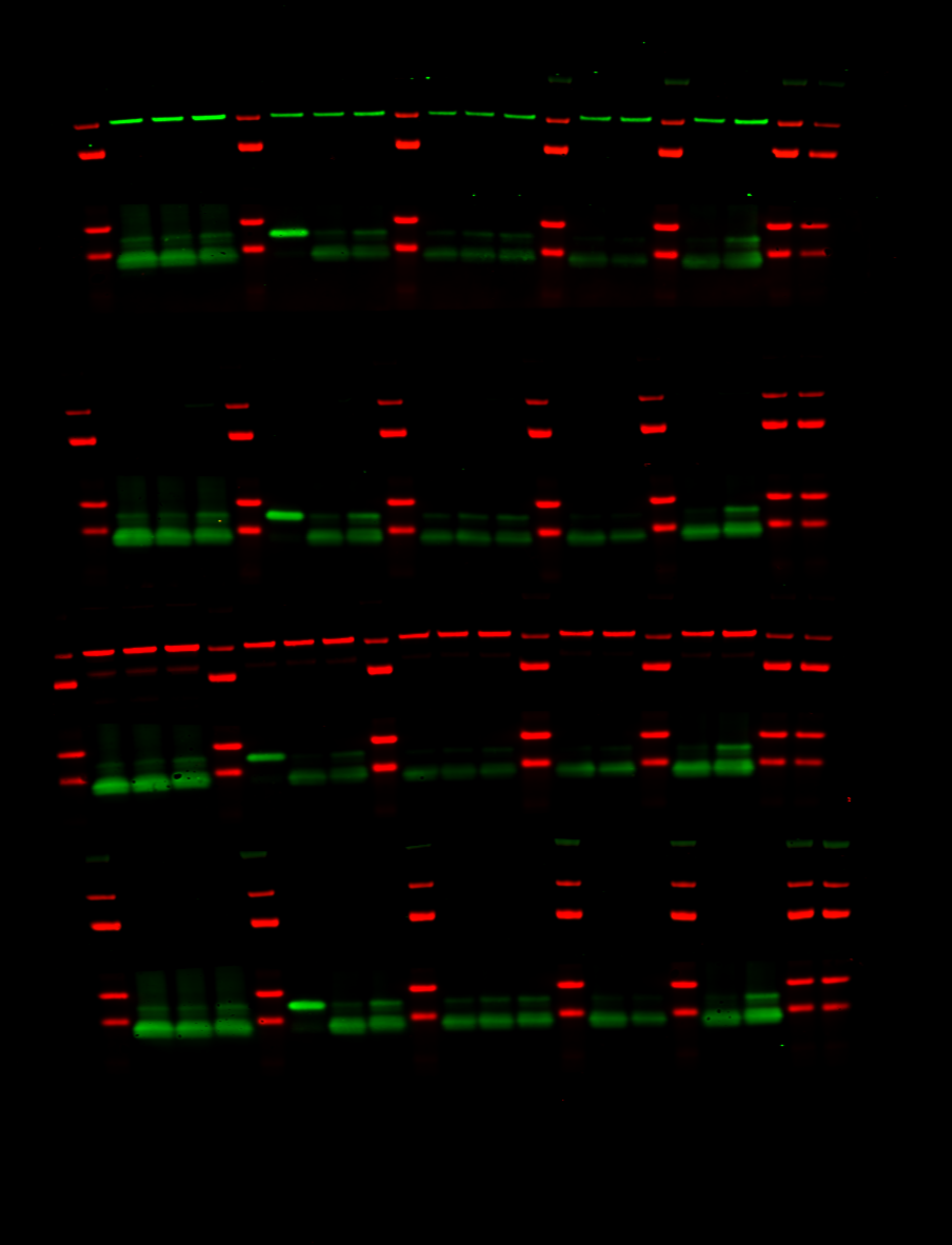

Supplement: Supplementary file 10 — Source Data [file 41467_2023_41891_MOESM10_ESM.zip › data/Fig 5/Fig5a:S4a uncropped blot tubulin PTMs.tif]

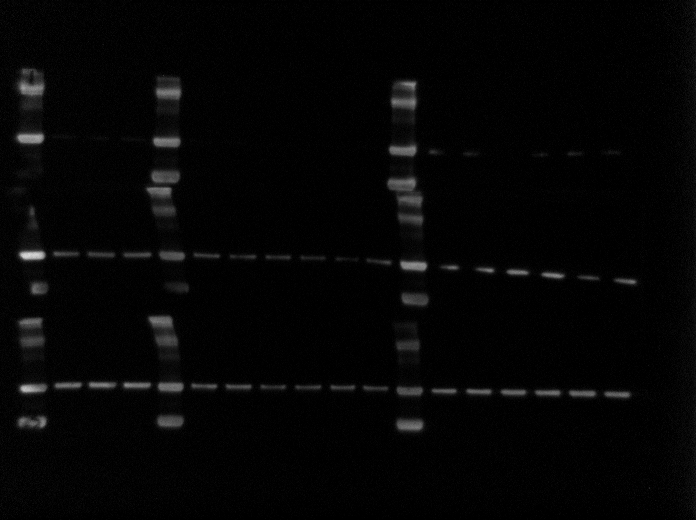

Supplement: Supplementary file 10 — Source Data [file 41467_2023_41891_MOESM10_ESM.zip › data/Fig 5/Fig5j:S4h uncropped blot Exp1 & Exp2.tif]

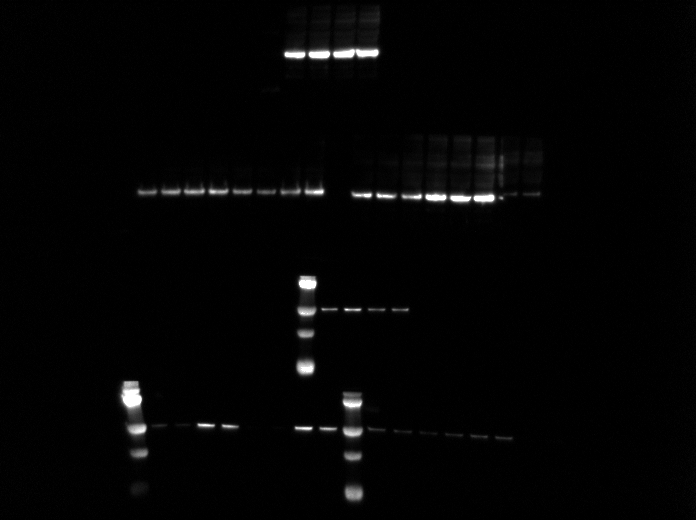

Supplement: Supplementary file 10 — Source Data [file 41467_2023_41891_MOESM10_ESM.zip › data/Fig 5/Fig5e:S4c uncropped blot Exp3.tif]

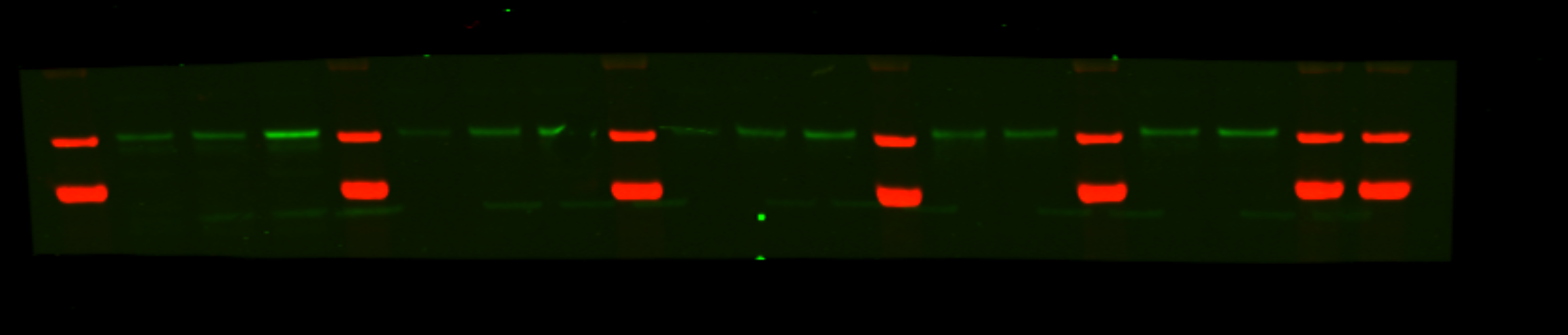

Supplement: Supplementary file 10 — Source Data [file 41467_2023_41891_MOESM10_ESM.zip › data/Fig 5/Fig5a:S4a uncropped blot acetylated tubulin.tif]

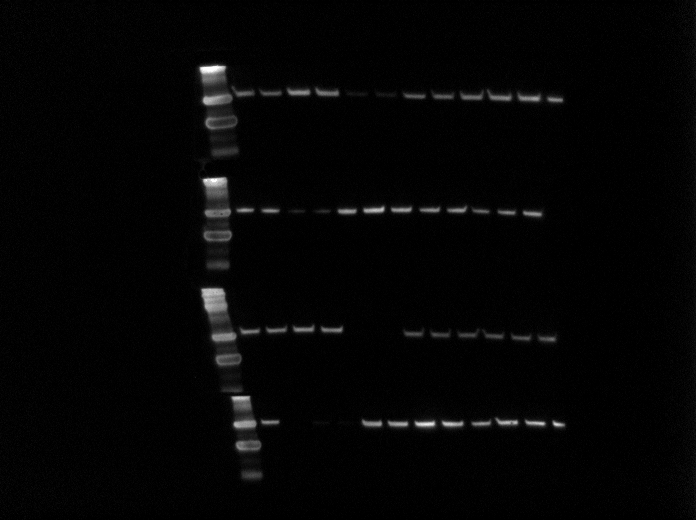

Supplement: Supplementary file 10 — Source Data [file 41467_2023_41891_MOESM10_ESM.zip › data/Fig 5/Fig5e:S4c uncropped blot Exp1&2 Sup&Pellet.tif]

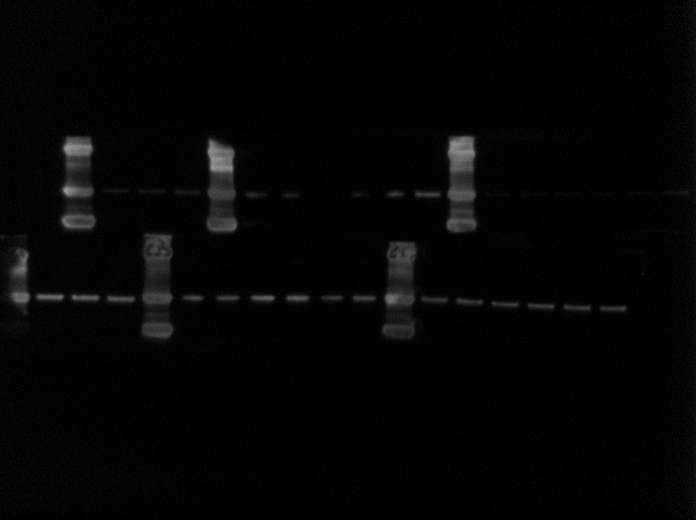

Supplement: Supplementary file 10 — Source Data [file 41467_2023_41891_MOESM10_ESM.zip › data/Fig 5/Fig5j:S4h uncropped blot Exp3.tif]
